# Alterations in the Expression of Proprotein Convertase Genes in Human Esophagus Squamous Cell Carcinomas

**DOI:** 10.32607/actanaturae.27437

**Published:** 2025

**Authors:** A. A. Komissarov, M. V. Zinovyeva, A. V. Sass, T. V. Vinogradova, S. I. Koshechkin, V. V. Demkin, I. B. Zborovskaya, S. V. Kostrov, I. V. Demidyuk

**Affiliations:** National Research Centre “Kurchatov Institute”, Moscow, 123182 Russian Federation; Shemyakin-Ovchinnikov Institute of Bioorganic Chemistry of the Russian Academy of Sciences, Moscow, 117997 Russian Federation; N.N. Blokhin National Medical Research Center of Oncology, Moscow, 115478 Russian Federation

**Keywords:** proprotein convertase, serine protease, cancer, gene expression, quantitative real-time PCR

## Abstract

Proprotein convertases (PCs) constitute an enzyme family that includes nine highly specific human subtilisin-like serine proteases. It is known that the PCs mRNA levels vary in tumors, and that these proteases are involved in carcinogenesis. Thus, PCs may be considered as potential markers for typing and predicting the course of the disease, as well as potential targets for therapy. We used quantitative real-time PCR to evaluate the expression levels of PC genes in the paired samples of tumor and adjacent normal tissues derived from 19 patients with esophageal squamous cell carcinomas. We observed a significant enrichment of *PCSK6*, *PCSK9*, *MBTPS1*, and *FURIN *mRNAs in the tumor tissue, which may be indication of the involvement of these PCs in the development and progression of esophageal cancers. Additionally, cluster analysis of PC expression alteration patterns in tumor compared to normal adjacent tissues (esophageal and previously analyzed lung tissue samples) revealed a limited set of scenarios for the changes in PC expression. These scenarios are implemented during malignant transformation of lung and esophagus cells, as well as, probably, the cells of other organs. These findings indicate that PC genes may be important markers of human cancers.

## INTRODUCTION

Proprotein convertases (PCs) are a family of highly specific mammalian subtilisin-like serine endopeptidases whose key function is processing various proteins and peptides [1, 2]. In humans, nine PC genes have been identified; the endogenous substrates of these proteases come in the form of numerous proteins and peptides, such as neuropeptide precursors, peptide hormones, growth and differentiation factors, receptors, and enzymes. It flows from this that PCs regulate a wide range of physiological processes, both in health and in disease. In particular, there is extensive evidence of an association between PCs and the development and progression of cancer.

PC substrates encompass a number of proteins involved in the progression of malignancies: cytokines, growth factors and their receptors, matrix metalloproteinases, and adhesion molecules (discussed in detail in [3, 4, 5]). There is ample evidence pointing to the fundamental role of PCs in tumor progression and metastasis, as well as the relationship between PC expression levels and patient survival [6, 7, 8, 9, 10, 11, 12]. All this suggests that data on PC expression levels may be used for typing and predicting the course of cancers, and that PCs themselves may serve as therapeutic targets.

Previously, we used quantitative PCR to demonstrate that PC expression in human lung malignancies was altered compared with that in the adjacent normal tissue. In this case, we, for the first time, found that the alterations in the expression occurred in a few scenarios: 80% of the analyzed samples formed three groups, each of which displayed a significantly altered expression of one of the three genes – *FURIN*,* PCSK1*, or *PCSK6 *(hereinafter, we use the Human Gene Nomenclature Committee (HGNC) guidelines for gene names, https://www.genenames.org, *Table 1*). We did not find any correlations between the identified groups and the available clinical data of patients [13]. However, the data we obtained may be indication that there exist unidentified properties of human lung tumors which are associated with one of the three identified scenarios of alterations in the PC expression.

**Table 1:** Genes and PCR kits used in the study

Protein	Gene (HGNC^*^)	Alternative protein names	PCR assay ID
Proprotein convertase subtilisin/kexin type 1	PCSK1	PC1/3, NEC1	Rn00567266_m1
Proprotein convertase subtilisin/kexin type 2	PCSK2	PC2, NEC2	Rn00562543_m1
Proprotein convertase subtilisin/kexin type 4	PCSK4	PC4	Rn00592006_m1
Proprotein convertase subtilisin/kexin type 5	PCSK5	PC5/6	Rn01450819_m1
Proprotein convertase subtilisin/kexin type 6	PCSK6	PACE4, SPC4	Rn00564475_m1
Proprotein convertase subtilisin/kexin type 7	PCSK7	PC7	Rn00570376_m1
Proprotein convertase subtilisin/kexin type 9	PCSK9	PC9, NARC-1	Rn01416753_m1
Membrane-bound transcription factor peptidase, site 1	MBTPS1	SKI-1/S1P	Rn00585707_m1
Furin	FURIN	PACE	Rn00570970_m1
Glyceraldehyde-3-phosphate dehydrogenase	GAPDH	G3PD	Rn01775763_g1

^*^HGNC – Human Gene Nomenclature Committee, https://www.genenames.org.

In this work, we studied esophageal malignancies in a way similar to that described above. Quantitative real-time PCR was used to analyze mRNA levels in tumor and adjacent normal tissues in samples obtained from 19 patients with esophageal squamous cell carcinoma. We found significantly increased expressions of the *PCSK6*, *PCSK9*, *MBTPS1*, and *FURIN* genes in tumor tissue, which may indicate the involvement of these PCs in the formation and progression of esophageal malignancies. We also performed a cluster analysis of PC expression alteration patterns in esophageal cancer samples and the previously analyzed lung cancer samples. As a result, a limited set of scenarios for PC expression alterations during malignant transformation of lung and esophageal cells and, probably, the cells of other organs, were identified.

## EXPERIMENTAL

Samples were collected in accordance with Federal Law No. 180 “On Biomedical Cell Products” (Order of the Ministry of Health of the Russian Federation No. 517n, Appendix 2, of August 11, 2017). The study protocol was approved by the Ethics Committee of the N.N. Blokhin National Medical Research Center of Oncology of the Ministry of Health of the Russian Federation (N.N. Blokhin NMRCO).

Esophageal tumor and adjacent normal tissue samples were obtained during surgery on patients with esophageal squamous cell carcinoma (stage II or III) at N.N. Blokhin NMRCO. Every patient provided written informed consent. The patients had not undergone chemotherapy or radiotherapy before surgery. Part of the samples was frozen in liquid nitrogen for subsequent RNA extraction; the other part was used for histological verification in the Department of Pathological Anatomy of Human Tumors of N.N. Blokhin NMRCO and graded in accordance with the TNM classification of the International Union Against Cancer [14]. According to the results of our histological examination, all malignant tumor tissue samples contained at least 70–80% of tumor cells. The tissue samples of the affected organ taken outside the pathological growth in each patient during surgery were used as control samples (conditional normal tissue).

Total RNA was isolated from the normal and tumor tissue samples that were frozen earlier and homogenized in liquid nitrogen. RNA was purified using guanidine isothiocyanate and phenol [15] and, then, an RNeasy Mini Kit (Qiagen, USA) according to the manufacturer’s protocol, followed by treatment with DNase I (Promega, USA). The concentration of isolated RNA was quantified by measuring absorption at 260 nm. The first cDNA strands were synthesized using a hexanucleotide primer (Promega, USA) and Powerscript reverse transcriptase (Clontech, USA).

Quantitative real-time PCR was performed on a CFX96 Touch device (Bio-Rad, USA) using predesigned primer and probe kits (Applied Biosystems, USA) (*Table 1*). The PCR program was as follows: 50°C for 2 min; 95°C for 10 min; then, 40 cycles: 95°C for 15 s and 60°C for 60 s. The reaction mixture volume was 20 μL (6 μL of deionized water, 4 μL of a 5X qPCRmix-HS PCR master mix (Eurogen, Russia), 5 μL of a primer and probe solution, and 5 μL of a sample). Each sample was analyzed in two independent experiments with duplicates. The Bio-Rad CFX Manager 3.1 software (Bio-Rad, USA) was used to process the PCR data and determine the cycle threshold (Ct) value.

PC mRNA levels were normalized to the mRNA levels of the reference gene *GAPDH *using the formula:

normalized_expression_of_a_PC_gene = 2Ct(GAPDH)–Ct(PC).

If expression of a PC gene was detected only in tumor or normal tissue, the normalized gene expression was calculated using the Ct value set to 42 for the missing sample.

Statistical data were processed using the R programming language (R Core Team (2023). R: A Language and Environment for Statistical Computing. R Foundation for Statistical Computing, Vienna, Austria. https://www.R-project.org/) and RStudio software (Posit team (2023). RStudio: Integrated Development Environment for R. Posit Software, PBC, Boston, MA. URL http://www.posit. co/). The differences in PC expression levels were evaluated using the paired Wilcoxon test. Cluster analysis of the samples was performed using Ward’s method with the Euclidean distance as a measure of the difference.

## RESULTS AND DISCUSSION

**Fig. 1:**
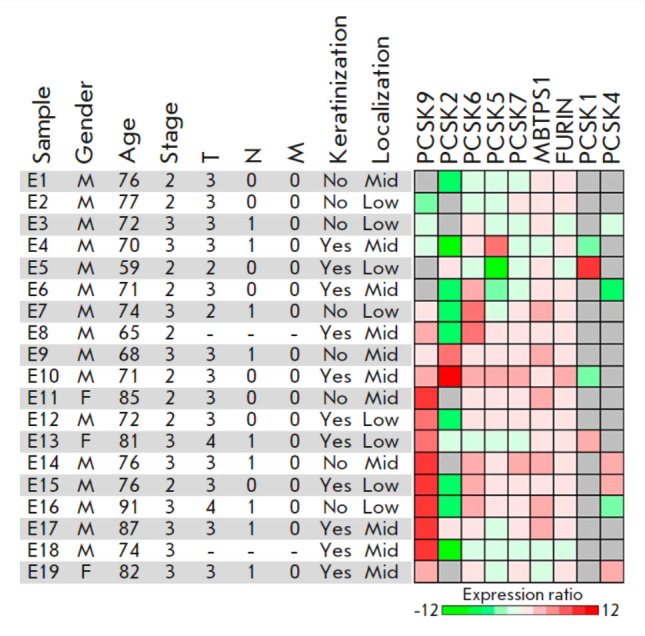
Sample characterization and heatmap representation of PC expression ratios in tumor compared to adjacent normal esophageal tissues. The heatmap is shown in the log2 scale. Gray cells indicate samples with undetectable gene expression in both tumor and normal tissues. Tumor localization is designated as the middle (mid) or lower (low) third of the esophagus. “–“, data are not available. T, N, and M denote tumor staging according to the TNM classification

In this study, the mRNA levels of all nine proprotein convertase (PC) genes in the 19 paired samples of human esophageal malignant and normal adjacent tissues were analyzed by quantitative real-time PCR (*Fig. 1*, Appendix *Table A1*). As expected, we detected expression of the *FURIN*, *PCSK5*, *PCSK6*, *PCSK7*, and *MBTPS1 *genes encoding ubiquitous enzymes in all or the vast majority of both tumor and normal tissue samples. *PCSK1*, *PCSK2*, *PCSK4*, and *PCSK9* expression is considered tissue-specific and atypical of esophageal tissues [16, 17, 18]. Indeed, *PCSK1 *mRNA was detected only in two tumor and two normal tissue samples and *PCSK4 *mRNA was identified in four tumor and two normal tissue samples. At the same time, *PCSK2 *expression, typical of neuroendocrine cells, was detected in 5 tumor and 11 normal tissue samples. The causes behind the atypical *PCSK2 *expression in esophageal tissues are unclear. We also detected* PCSK9 *expression, which is normally observed mainly in liver, kidney, cerebellum, and small intestine cells, in 15 tumor and 9 normal esophageal tissue samples. However, this result was not unexpected, because *PCSK9 *expression in esophageal tumors was detected previously [19]. The possible causes of this phenomenon will be discussed below.

**Fig. 2:**
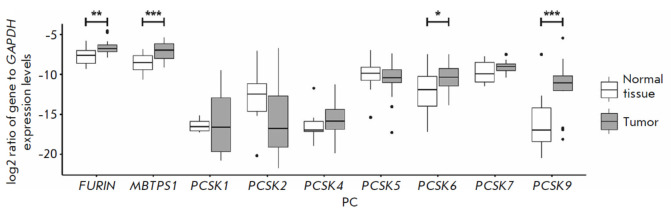
PC expression levels in esophageal tumor and normal samples. PC mRNA levels were analyzed using quantitative real-time PCR as indicated in the Experimental section and then normalized to the mRNA levels of the *GAPDH *reference gene. The significance of the differences in normalized PC mRNA levels between tumor and adjacent normal tissues was analyzed using the Wilcoxon paired test. *, *p* < 0.05; **, *p* < 0.01; ***, *p* < 0.001

Comparison of PC expression levels revealed that* PCSK6*, *PCSK9*, *MBTPS1*, and *FURIN *expressions in the esophageal tumor tissue were statistically significantly higher than those in the normal adjacent tissue (*Fig. 2*). In this case, expressions of the *PCSK9 *and* PCSK6 *genes proved upregulated most significantly, approximately 175- and 10-fold higher, respectively, on average. Expressions of the *MBTPS1 *and *FURIN* genes were upregulated moderately, approximately 4- and 3-fold higher, respectively, on average. A significant increase in PCSK9 expression at the protein level in the esophageal tumor tissue compared with that in normal tissue was previously detected in a study by Wang et al.; in that case, patients with high PCSK9 expression levels in tumors had a lower survival likelihood [19]. A study by Ito et al. revealed that a high anti-PCSK9 antibody titer in the blood of esophageal cancer patients correlated with a higher survival likelihood in the postoperative period [20]. We could not find any studies that reported increased *MBTPS1 *expression in esophageal tumors. Regarding the *PCSK6* and *FURIN *genes, their association with oncological diseases has been established in many studies [3, 7, 8, 9, 11, 21, 22, 23, 24, 25, 26].

It is noteworthy that two PC genes with upregulated expression in esophageal cancer cells are involved in lipid homeostasis. Thus, *PCSK9 *encodes a key regulator of blood low-density lipoprotein–cholesterol complex levels and the product of the *MBTPS1* gene plays an important role in the regulation of cholesterol, lipid, and fatty acid synthesis. To date, a substantial amount of data has been gathered indicating the fundamental role of lipid metabolism and biosynthesis in the development and progression of cancers [27]. In this context, our findings suggest that PCSK9 and MBTPS1 may be involved in the progression of human esophageal cancer through the regulation of lipid homeostasis in the tumor.

Recently, the role of PCSK9 in immune response evasion by tumor cells has begun to be actively studied. For example, PCSK9 inhibition was shown to increase the effectiveness of immunotherapy against a number of cancers (for details, see [28]). However, the first line of treatment for stage II and III esophageal cancer is chemoradiotherapy without additional immune drugs. However, a recent study, CheckMate 577, reported that the combination of chemoradiotherapy and the neoadjuvant drug Nivolumab approximately doubled the median relapse-free survival time compared with chemoradiotherapy alone [29]. Nivolumab is a monoclonal antibody from the group of immune checkpoint inhibitors; it specifically inactivates the PD-1 protein on the cell surface. The PD-1 protein plays an important role in the inhibition of immune responses through suppression of T cell activity, inducing apoptosis of activated antigen-specific T cells and, conversely, inhibition of apoptosis of anti-inflammatory regulatory T cells [30]. Recently, mouse models were used to show an inverse relationship between the efficacy of anti-PD-1 therapy and PCSK9 expression levels, as well as a significant increase in the antitumor effect by combined inhibition of PD-1 and PCSK9 [31]. These data, along with the significantly increased PCSK9 expression in tumor tissue, found in our study, indicate that simultaneous blockade of PD-1/PCSK9 may be considered a promising approach to improve the efficacy of human esophageal tumor therapy.

**Fig. 3:**
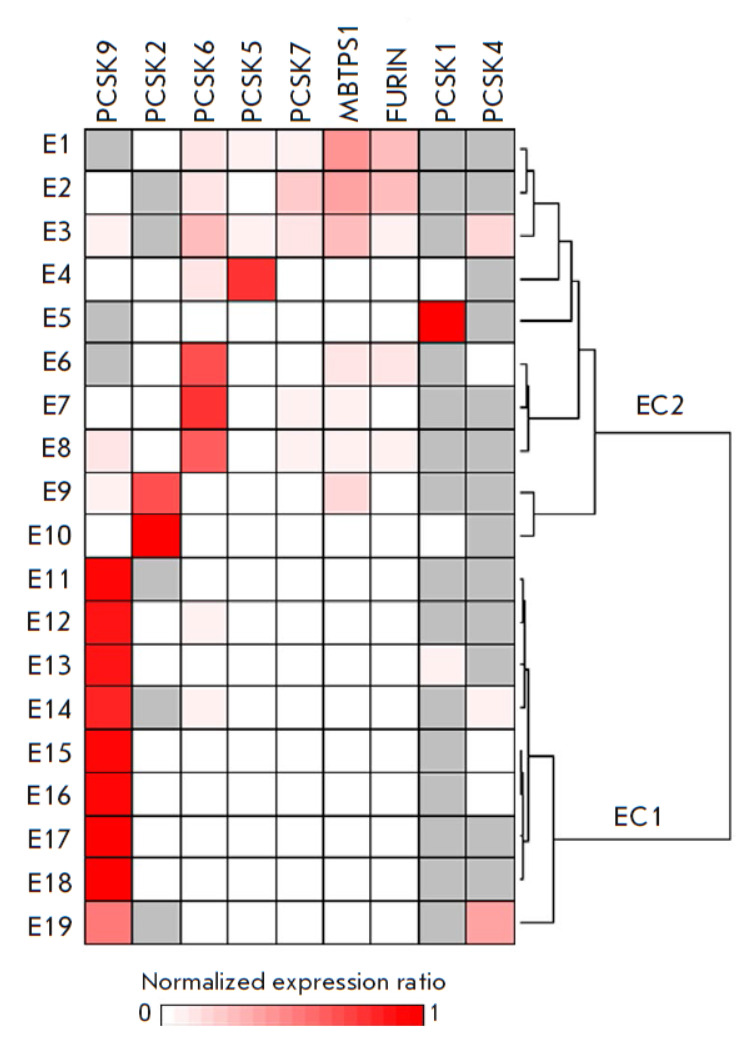
Cluster analysis of PC expression patterns in esophageal samples. Normalized PC mRNA level ratios in tumor compared to normal tissues were additionally row-normalized and presented as a heatmap in fractions of one. Gray cells indicate samples with undetectable gene expression in both tumor and normal tissues. Row-normalized data were clustered as indicated in the Experimental section. The dendrogram indicates the distance between samples. The identified clusters are designated as EC1 and EC2

Cluster analysis of PC expression alteration patterns in esophageal tumor compared to normal tissue samples revealed two groups of samples (*Fig. 3*). The first group included 9 (47%) samples with significantly upregulated *PCSK9 *expression (*Fig. 3*, cluster EC1). The second group was less homogeneous (*Fig. 3*, cluster EC2) and included samples with significantly increased expressions of the *PCSK6 *(three samples, 16%), *PCSK2* (two samples, 10%), *PCSK1 *(one sample, 5%), and *PCSK5 *(one sample, 5%) genes, or no significant changes in any individual gene (three samples, 16%). The PC expression alteration patterns identified in esophageal tumors differed from those we had identified previously in lung tumors [13]. For example, esophageal tumors lacked clusters with predominant alterations in the *FURIN *and *PCSK1 *expressions, which included most of the lung tumor samples (18 of 30, 60%), whereas lung tumors lacked the cluster with predominant alteration in *PCSK9 *expression, which was the most abundant in esophageal tumors. Meanwhile, samples with altered *PCSK6 *expression were detected in both nosologies. Our findings indicate that the PC expression alterations in tumor tissue compared to adjacent normal tissue differ across tumor types, but that there is a limited set of scenarios for PC expression alterations in each case.

**Fig. 4:**
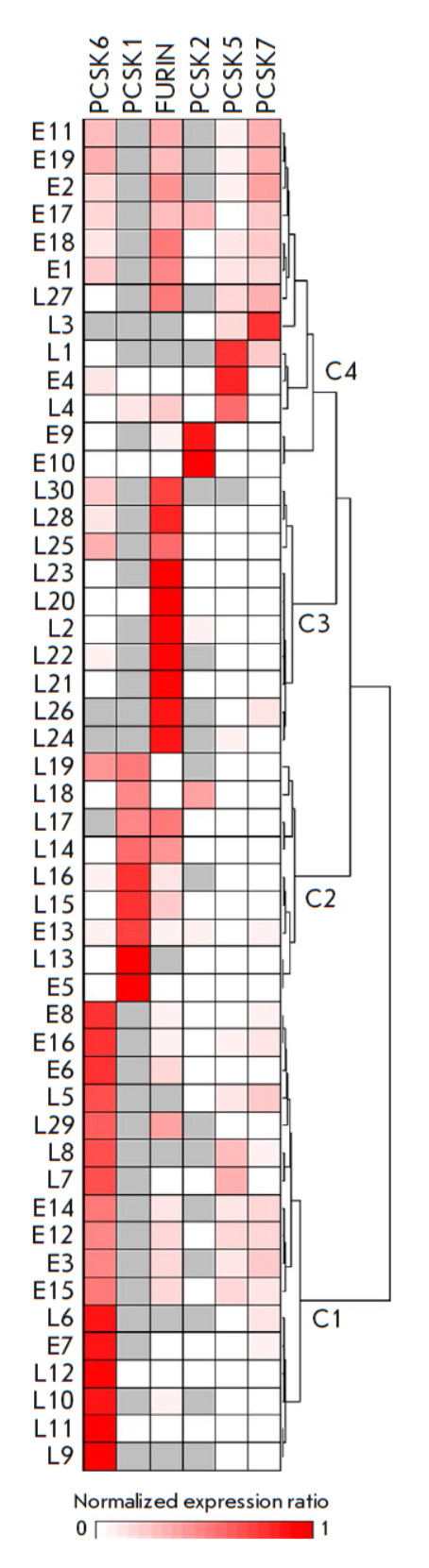
Cluster analysis of the expression patterns of the six key PC genes in esophageal and lung tumors. Normalized PC mRNA level ratios in tumor compared to normal tissues were additionally row-normalized and presented as a heatmap in fractions of one. Gray cells indicate samples with undetectable gene expression in both tumor and normal tissues. Lung tumors are designated as L1–L30, and esophageal samples are designated as E1– E19. Row-normalized data were clustered as indicated in the Experimental section. The dendrogram indicates the distance between samples. The identified clusters are designated as C1–C4

Investigation of PC functions, in particular using gene knockout rodents, has revealed that most of these enzymes have overlapping substrate specificity. PCSK1 and PCSK2 were shown to act on common substrates, and the substrate specificity overlap of the FURIN, PCSK5, PCSK6, and PCSK7 proteins is so broad that they are able to partially compensate for each other’s lack in tissues [32, 33]. At the same time, the PCSK4, PCSK9, and MBTPS1 enzymes act on a narrow range of quite specific substrates. In this regard, the functioning of six PCs (PCSK1, PCSK2, PCSK5, PCSK6, PCSK7, and FURIN) may potentially be considered as a single protein processing system. Cluster analysis of the expression alteration patterns of these six PCs in the total sample of lung and esophageal tumors revealed four sample groups (*Fig. 4*). Three groups included almost three quarters of the samples (*n *= 36, 73.5%) and were characterized by a predominant change in the expression of one PC gene: *PCSK6*, *PCSK1*, or *FURIN *(the groups correspond to clusters C1, C2, and C3, respectively, in *Fig. 4*). Group C3 consisted exclusively of lung tumor samples, whereas groups C1 and C2 included samples of both nosologies. The fourth group that corresponded to cluster C4 in *Fig. 4* was more heterogeneous and included the remaining quarter of the samples (*n *= 13, 26.5%). In this group, three subgroups may be distinguished; of these, two include five samples with significantly altered expression of the *PCSK2 *or* PCSK5 *gene. However, most samples (8 out of 13) belong in the third subgroup, characterized by increased expression of the *PCSK6*, *PCSK7*, and *FURIN *genes with a lack of *PCSK1 *expression. We did not find statistically significant correlations between the identified sample groups and available clinical data from the patients. Nevertheless, the obtained data may be indication that there is a limited number of scenarios for PC expression alterations during the malignant transformation of cells and the genesis of lung and esophageal tumors, as well as, possibly, tumors of other nosologies.

Unfortunately, the available information may only be suggestive of the nature of the differences between the identified sample groups. For example, high* PCSK6 *expression in group C1 may indicate active restructuring of the tumor microenvironment and, thus, correlate with the invasiveness and/or metastatic activity of the tumor [25, 26]. Increased *PCSK1 *expression in group C2 may be related to the possible origin of the tumor from neuroendocrine cells [34, 35]. Increased levels of *FURIN *mRNA in group C3 may be associated with active expression and processing of the insulin-like growth factor 1 receptor (IGF-1R) in tumors, indicating their increased aggressiveness [36]. Obviously, further research is required to elucidate the causes underlying the identified scenarios of PC expression alterations. Probably, more detailed investigation of a larger batch of samples from different nosologies is required, including analysis of additional clinical characteristics, in particular, patients’ resistance to drugs, relapse rates, and patients’ survival time.

It should also be noted that this study was conducted on a relatively small number of samples. Therefore, the changes in the expression of PC genes and the groups identified during cluster analysis need confirmation with a larger sample size. Nevertheless, the samples analyzed here include lung and esophageal tumors, representing two independent and quite heterogeneous groups, which, however, are characterized by similar patterns in PC expression alterations. In this regard, these patterns are likely to be quite pronounced and, thus, can be detected even on a small sample size, which makes mRNAs of PC genes potentially important tumor markers.

## CONCLUSION

Our analysis revealed increased *PCSK6*, *PCSK9*,* MBTPS1*, and *FURIN *expressions in human esophageal tumors. This indicates the potential involvement of these PCs in the development and progression of esophageal malignancies. In this case, the role of *PCSK9* and *MBTPS1* in the pathological process is probably associated with the involvement of the protein products of these genes in lipid metabolism and/or immune response evasion by tumor cells. We found that alterations in PC expression in esophageal and lung tumors follow a limited set of similar scenarios. This may be indicative of common mechanisms of malignant transformation of lung and esophageal cells and, possibly, tumors of other localizations.

## Abbreviations

IGF-1R: insulin-like growth factor 1 receptor
PC: proprotein convertase
